# Kaempferol Can Reverse the 5-Fu Resistance of Colorectal Cancer Cells by Inhibiting PKM2-Mediated Glycolysis

**DOI:** 10.3390/ijms23073544

**Published:** 2022-03-24

**Authors:** Haili Wu, Jin’e Du, Chenglu Li, Hanqing Li, Huiqin Guo, Zhuoyu Li

**Affiliations:** 1 College of Life Science, Shanxi University, Taiyuan 030006, China; whl@sxu.edu.cn (H.W.); 202023118002@email.sxu.edu.cn (J.D.); 201923106001@email.sxu.edu.cn (C.L.); 202123118004@email.sxu.edu.cn (H.L.); 2 The Key Laboratory of Chemical Biology and Molecular Engineering of Ministry of Education, Institute of Biotechnology, Shanxi University, Taiyuan 030006, China; 201913002002@email.sxu.edu.cn

**Keywords:** kaempferol, 5-Fu resistance, miR-326, PKM2, aerobic glycolysis, colorectal cancer

## Abstract

Resistance to 5-Fluorouracil (5-Fu) chemotherapy is the main cause of treatment failure in the cure of colon cancer. Therefore, there is an urgent need to explore a safe and effective multidrug resistance reversal agent for colorectal cancer, which would be of great significance for improving clinical efficacy. The dietary flavonoid kaempferol plays a key role in the progression of colorectal cancer and 5-Fu resistance. However, the molecular mechanism of kaempferol in reversing 5-Fu resistance in human colorectal cancer cells is still unclear. We found that kaempferol could reverse the drug resistance of HCT8-R cells to 5-Fu, suggesting that kaempferol alone or in combination with 5-Fu has the potential to treat colorectal cancer. It is well known that aerobic glycolysis is related to tumor growth and chemotherapy resistance. Indeed, kaempferol treatment significantly reduced glucose uptake and lactic acid production in drug-resistant colorectal cancer cells. In terms of mechanism, kaempferol promotes the expression of microRNA-326 (miR-326) in colon cancer cells, and miR-326 could inhibit the process of glycolysis by directly targeting pyruvate kinase M2 isoform (PKM2) 3′-UTR (untranslated region) to inhibit the expression of PKM2 or indirectly block the alternative splicing factors of PKM mRNA, and then reverse the resistance of colorectal cancer cells to 5-Fu. Taken together, our data suggest that kaempferol may play an important role in overcoming resistance to 5-Fu therapy by regulating the miR-326-hnRNPA1/A2/PTBP1-PKM2 axis.

## 1. Introduction

Human colorectal cancer is a kind of gastrointestinal cancer. According to the latest global cancer burden data for 2020 released by the International Agency for Research on Cancer of the World Health Organization, the incidence and mortality of colorectal cancer ranks third (10% of total cases) and second (9.4% of the total cancer deaths) among all malignant tumors [1]. At present, the main methods for the treatment of colon cancer are surgery, chemotherapy, radiotherapy, immunotherapy and targeted therapy, while in long-term clinical use of chemotherapeutic drugs, patients will have toxic side effects and drug resistance [2]. 5-Fluorouracil (5-Fu), oxaliplatin (L-OHP), and vincristine (VCR) are common chemotherapeutic agents which are widely used to treat colorectal cancer [3,4]. Although chemotherapeutic agents have improved the survival rate of colorectal cancer patients, resistance to chemotherapy remains a major hurdle to obtain a cure for this malignancy. Traditional chemical reversal is limited in clinical application due to the single mechanism and serious side effects; quinine, cyclosporine A and verapamil, for instance, are always used in large doses and they can cause serious adverse cardiovascular reactions [5,6]. Therefore, exploring safe and effective drug resistance reversal agents for human colorectal cancer is of great importance for the improvement of clinical effects.

The occurrence of a tumor is accompanied by a change in the tumor’s microenvironment. Aerobic glycolysis is common in most cancer cells, which allows them to produce energy even in the presence of oxygen, followed by lactic acid fermentation, known as the Warburg effect [7]. More and more evidence shows that inhibition of glycolysis can effectively induce apoptosis in drug resistant cells, suggesting that targeting glycolysis may be a new strategy to overcome drug resistance [8]. The pyruvate kinase M2 isoform (PKM2) is one of the most important regulatory factors in glycolysis, and it plays an important role in the process of drug resistance of tumor cells [9]. There are four subtypes of pyruvate kinase in mammals, which are encoded by two different genes. Pyruvate kinase M1 isoform (PKM1) and M2 isoform (PKM2), encoded by PKM gene, are two common pyruvate kinase subtypes. They have the same gene coding length and alternating mutually exclusive exons, and can encode 56 amino acid residues. Under the action of shear factors, mRNA transcribed by PKM can form PKM1 containing exon 9, which is usually expressed in normal tissues, and PKM2 containing exon 10, mainly expressed in embryonic cells and tumor cells. It has been found that the splicing factors of the PKM gene mainly include three heterogeneous nuclear proteins of polypyrimidine-tract-binding protein (PTBP1), heterogeneous nuclear ribonucleoprotein A1 (hnRNPA1) and heterogeneous nuclear ribonucleoprotein A2 (hnRNPA2), which release exon 10 by binding to exon 9, thus promoting the expression of PKM2 and inhibiting the expression of PKM1 [10]. It is reported that depletion or inhibition of PKM2 can re-sensitize cisplatin-resistant bladder cancer cells [11]. In addition, PKM2 gene silencing enhanced the effects of docetaxel and cisplatin in vitro and in lung cancer xenotransplantation models [12].

Recently, natural phenolic extraction has drawn more and more attention in overcoming drug resistance in human cancer cells. Shakibaei et al. reported that curcumin combined with 5-Fu can restore the sensitivity of human colon cancer cells to 5-Fu and reverse their multidrug resistance [13]. Research by Leslie et al. found that the natural phenolic compounds ellagic acid and Schisandra chinensis have therapeutic potential to overcome the multidrug resistance of cancer [14]. Nabekura and colleagues also showed that capsaicin could increase the intracellular P-gp substrates by inhibiting chemotherapeutic drug efflux transporters [15]. In fact, studies have shown that some secondary metabolites contained in medicine and food homologous plants play an important role in improving the effect of chemotherapy and preventing multidrug resistance. These compounds not only kill cancer cells, but also restore drug sensitivity [16]. Therefore, there is an urgent need to develop a natural compound with high efficiency and low side effects as a multidrug resistance reversal agent for colorectal cancer to improve the effectiveness of chemotherapy and overcome drug resistance.

Here, we explored the mechanism of kaempferol in reversing the drug resistance of colon cancer, and proved for the first time that kaempferol can up-regulate the expression of microRNA-326 (miR-326); we also showed that miR-326 inhibits the expression of PKM2 by directly targeting the 3′-UTR (untranslated region) of PKM2 or indirectly blocking the alternative splicing factor of PKM mRNA, and re-sensitizes 5-Fu drug-resistant colon cancer cells to 5-Fu by antagonizing glycolysis. The current research results emphasize that the exploration of safe and effective multidrug resistance reversal agents for colorectal cancer and its mechanism is of great significance to improve the clinical efficacy.

## 2. Results

### 2.1. Identification of the 5-Fu Resistance of HCT8-R Cells

**Figure 1:**
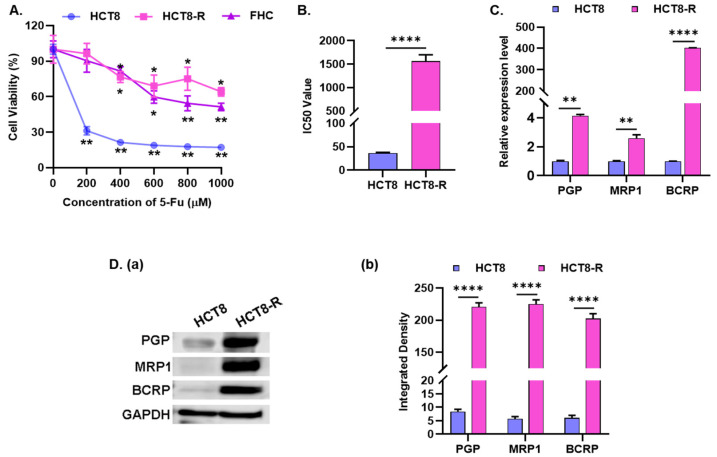
Identification of the 5-Fu resistance of HCT8-R cell. (**A**) The cell viability of FHC, HCT8-R and the parental cell line HCT8 was determined by CCK-8 assay upon 5-Fu treatment. Data represent means ± SD from three independent experiments. (**B**) The IC_50_ value of 5-Fu in HCT-8 and HCT8-R cells. (**C**) qPCR assay was performed to test the mRNA expression of PGP (p-glycoprotein), MRP1 (multidrug resistance protein 1) and BCRP (breast cancer resistance protein). (**D**) Western blot analysis was carried out to detect the protein level of PGP, MRP1 and BCRP. GAPDH was used as a reference protein for equal loading. (**a**) A representative result from three independent experiments was shown. (**b**) The relative protein expression levels were analyzed using Image J. * *p* < 0.05, ** *p* < 0.01, **** *p* < 0.0001.

Tumor drug resistance generally means that after repeated contact with chemotherapeutic drugs, the sensitivity of tumor cells to chemotherapeutic drugs decreases or even disappears, resulting in reduced or ineffective efficacy of drugs on cancer cells. In order to determine the drug resistance of the HCT8-R cell line to 5-Fu, the effects of different concentrations of 5-Fu on the viability of human normal colorectal mucosal cell FHC, HCT8 and HCT8-R cells were detected. The results indicated that compared with the control group with 0 μM concentration, 5-Fu significantly inhibited the proliferation of HCT8 parent cells rather than HCT8-R-resistant cells and had poor cytotoxic activity for human normal colorectal mucosal cell FHC (IC_50_ = 937.9 ± 1.38 μM) (Figure 1A). Moreover, the IC_50_ value in HCT8-R cells was 1563 ± 1.47 µM, which was 42.6-fold higher than that of HCT8 cells (IC_50_ = 36.7 ± 1.16 µM), that is, the drug resistance index was 42.6 (Figure 1B). ATP-binding cassette (ABC) transporters such as p-glycoprotein (PGP, ABCB1), multidrug resistance protein 1 (MRP1, ABCC1) and breast cancer resistance protein (BCRP, ABCG2) have been reported to be closely related to drug resistance, and they are usually overexpressed in cancer patients who are not responsive to chemotherapy drugs. Therefore, the expression of the above proteins was investigated by qPCR and Western blot. Indeed, both mRNA (Figure 1C) and protein levels (Figure 1D) were significantly up-regulated in HCT8-R cells. These data suggest that HCT8-R cells do have a strong resistance to 5-Fu, which can be used as a colorectal cancer drug resistance cell model for follow-up experiments.

### 2.2. Kaempferol Reverses the 5-Fu Resistance of HCT8-R Cells

**Figure 2:**
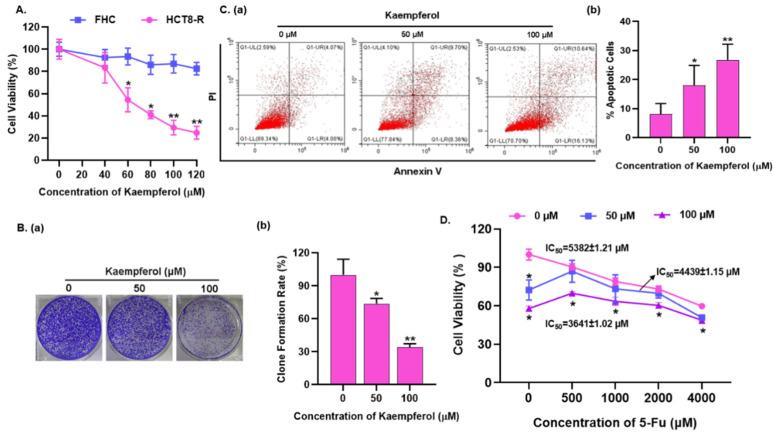
Kaempferol reverses the 5-Fu resistance in HCT8-R cells. (**A**) HCT8-R and FHC cells were treated with increasing concentrations of kaempferol for 48 h, and the cell viability was determined by CCK-8 assay. The blank control group (0 μM) represented 0.05% (*v/v*) DMSO (dimethyl sulfoxide). Data represent means ± SD from three independent experiments. (**B**) HCT8-R cells were seeded into a 6-well plate at a density of 5 × 10^3^/well and treated with various doses of kaempferol for one week; then the cell clone formation ability was explored. (**a**) Representative graphs are shown. (**b**) The quantitative analysis of colony formation. (**C**) Analysis of apoptosis in HCT8-R cells treated with kaempferol for 48 h using flow cytometry. (**a**) Representative graphs are shown. (**b**) The apoptotic rate is shown in bar format. (**D**) HCT8-R cells were pretreated with different concentrations of kaempferol for 24 h, then treated with 5-Fu for another 24 h, and the cell survival rate was determined by CCK-8 assay. * *p* < 0.05, ** *p* < 0.01.

In order to detect the reversal ability of kaempferol on drug resistant cell line HCT8-R, the inhibitory effect of kaempferol on HCT8-R cells was detected by CCK-8 assay at first. It was found that compared with blank control group, kaempferol treatment significantly suppressed the proliferation of HCT8-R (IC_50_ = 70.39 ± 1.15 μM) in a dose-dependent manner but had no significant inhibitory effect on FHC (IC_50_ = 1140 ± 1.15 μM) in the effective concentration range (Figure 2A). Accordingly, the clonogenic ability of HCT8-R was largely suppressed upon kaempferol treatment, and the inhibitory effect was up to approximately 70% in the concentration of 100 μM (Figure 2B). Moreover, kaempferol could largely induce the apoptosis of HCT8-R. As shown in Figure 2C, the apoptotic rates were 18.06 ± 3.66 and 26.77 ± 5.46 in HCT8-R cells treated with 50 and 100 μM kaempferol, respectively. To further assess the reversal effect of kaempferol on HCT8-R cells, after HCT8-R cells were pretreated with different concentrations of kaempferol for 24 h, various concentrations of 5-Fu were added to treatment for another 24 h, and a CCK-8 assay was finally employed to assess the cell viability. It was found that 50 μM and 100 μM kaempferol pre-treatment significantly enhanced the chemotherapy sensitivity of HCT8-R (Figure 2D). The IC_50_ of 5-Fu decreased from 5382 ± 1.21 μM to 4439 ± 1.15 μM and 3641 ± 1.02 μM, respectively. It is well known that a reversal multiple equal to 1 or more indicates that the active molecule can reverse drug resistance, so that the reversal multiple of kaempferol was calculated. According to the formula of resistance reversal fold = (IC_50_ of HCT8-R to 5-Fu before kaempferol treatment) / (IC_50_ of HCT8-R to 5-Fu after kaempferol treatment), the reversal multiples of 50 μM and 100 μM kaempferol to 5-Fu were 1.21 and 1.48 respectively. To sum up, our results show that kaempferol can effectively reverse the 5-Fu resistance of HCT8-R cells.

### 2.3. Kaempferol Promotes 5-Fu Sensitivity by Inhibiting Glycolysis

**Figure 3:**
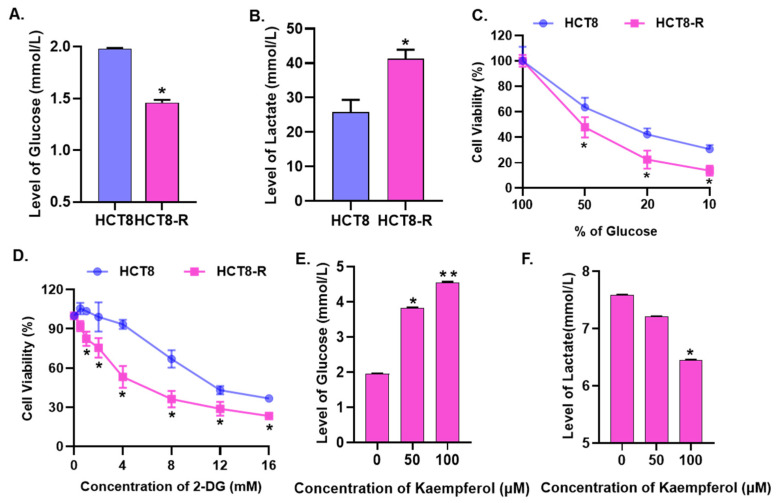
Kaempferol promotes 5-Fu sensitivity by inhibiting glycolysis. (**A**) Analysis of glucose content in HCT8 and HCT8-R cell culture media. (**B**) The content of lactate was detected in HCT8 and HCT8-R cell culture media. (**C**) After HCT8 and HCT8-R cells were cultured in glucose deprivation medium containing 10%, 20%, 50% and 100% glucose for 24 h, a CCK-8 assay was used to detect cell survival. Data represent means ± SD from three independent experiments. (**D**) After HCT8 and HCT8-R cells were treated with various concentrations of 2-DG (2-deoxy-d-glucoside) (0 mM, 0.5 mM, 1 mM, 2 mM, 4 mM, 8 mM,12 mM and 16 mM) for 48 h, a CCK-8 assay was used to detect cell survival. Data represent means ± SD from three independent experiments. (**E**) After HCT8-R cells were treated with kaempferol for 48 h, the content of glucose in the culture medium was detected. (**F**) After HCT8-R cells were treated with kaempferol for 48 h, the content of lactate in the culture medium was detected. * *p* < 0.05, ** *p* < 0.01.

Drug-resistant cells are often accompanied by enhanced aerobic glycolysis, suggesting that targeted glycolysis is a potential new strategy to overcome drug resistance. We thereby assessed glycolysis in HCT8-R cells and the parental cells. It was observed that the glucose content of HCT8-R cell culture medium exhibited a significant decrease (Figure 3A), and the lactate content an increase (Figure 3B), compared with HCT8 cells, indicating that HCT8-R cells have higher glycolysis activity. Additionally, we compared the cell viability of HCT8-R and HCT8 cells under the condition that the glycolysis was suppressed by treatment with glucose deprivation medium or 2-deoxy-d-glucoside (2-DG), which is an inhibitor of glucose synthesis that blocks glycolysis flux by inhibiting the activities of hexokinase and glucose phosphate isomerase. The results show that HCT8-R cells were more sensitive to glucose deficiency than HCT8 cells at the same concentration (Figure 3C). Similarly, the same concentration of 2-DG treatment displayed decreased cell viability in HCT8-R cells compared with HCT8 cells (Figure 3D). Based on the above observations, we investigated the effect of kaempferol on glycolysis level in HCT8-R cells. The results show that kaempferol treatment dramatically increased the content of glucose in HCT8-R cell culture medium (Figure 3E) and decreased the content of lactate (Figure 3F), suggesting that kaempferol might promote the 5-Fu sensitivity by inhibiting glycolysis.

### 2.4. PKM2 Mediates Kaempferol, Increasing the HCT8-R’s Sensitivity to 5-Fu

**Figure 4:**
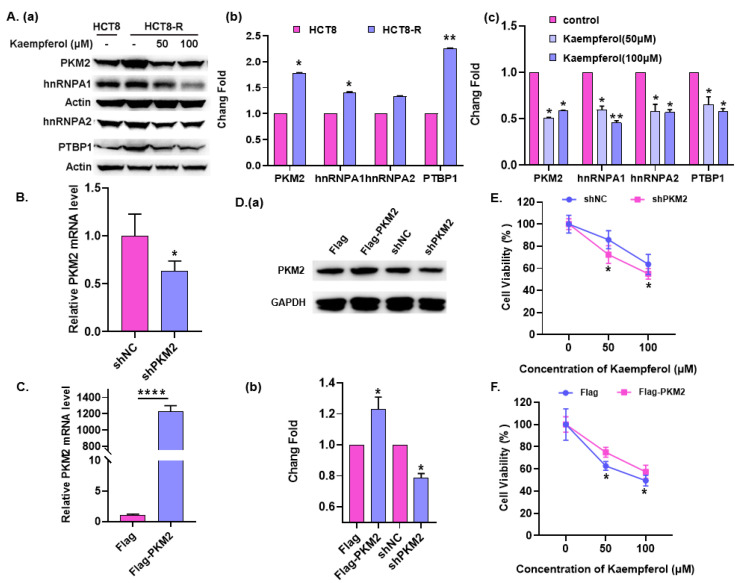
PKM2 mediates kaempferol, increasing the HCT8-R’s sensitivity to 5-Fu. (**A**) The protein expression of PKM2 (pyruvate kinase M2 isoform) and hnRNPA1/A2 (heterogeneous nuclear ribonucleoprotein A1/A2)/PTBP1 (polypyrimidine-tract-binding protein) in HCT8 and HCT8-R cells was analyzed by Western blot. (**a**) A representative result from three independent experiments is shown. (**b**,**c**) The densitometry analysis of relative protein expression is shown in a histogram. (**B**,**C**) qPCR was used to analyze the expression of PKM2 mRNA in HCT8-R cells after knockdown (**B**) and over-expression (**C**) of PKM2. (**D**) Western blot was used to analyze the expression of PKM2 protein in HCT8-R cells after overexpression or knockdown of PKM2. (**a**) A representative result from three independent experiments is shown. (**b**) The densitometry analysis of relative protein expression is shown in a histogram. (**E**) After transfecting with shNC or shPKM2, the HCT8-R cells were cultured in medium containing kaempferol for 48 h, and a CCK-8 assay was used to detect cell survival. (**F**) After transfecting with Flag or Flag-PKM2, the HCT8-R cells were cultured in medium containing kaempferol for 48 h, and a CCK-8 assay was used to detect cell survival. Data represent means ± SD from three independent experiments. * *p* < 0.05, ** *p* < 0.01, **** *p* < 0.0001.

The above results suggest that kaempferol may promote the sensitivity of 5-Fu by inhibiting glycolysis. PKM2 is one of the most important regulators of glycolysis, and it has been reported as playing a crucial role in the drug resistance of tumor cells. In order to find out how kaempferol reverses aerobic glycolysis in HCT8-R drug-resistant cell lines, we detected the expression of the key rate-limiting enzyme in glycolysis, PKM2, and its three splicing factors in HCT8 and HCT8-R cells. It was found that PKM2 protein levels were markedly increased in HCT8-R cells compared to HCT8 cells (Figure 4(Aa,Ab)). Interestingly, kaempferol treatment largely strained the expression of PKM2 (Figure 4(Aa,Ac)). With the decrease in PKM2 expression in HCT8-R cells treated with kaempferol, we speculate that kaempferol may re-sensitize HCT8-R cells to 5-Fu by inhibiting the expression of PKM2. To this end, we overexpressed and knocked down PKM2 in HCT8-R cells, and then cultured them with different concentrations of kaempferol. Through the CCK-8 experiment, it was found that PKM2 knockdown and overexpression decreased and increased the cell viability of HCT8-R, respectively, at the same concentration of kaempferol (Figure 4B–F). These results suggest that PKM2 plays an important role in 5-Fu resistance and mediates kaempferol, increasing the HCT8-R’s sensitivity to 5-Fu.

### 2.5. Kaempferol Regulates PKM2 Expression through miR-326

**Figure 5:**
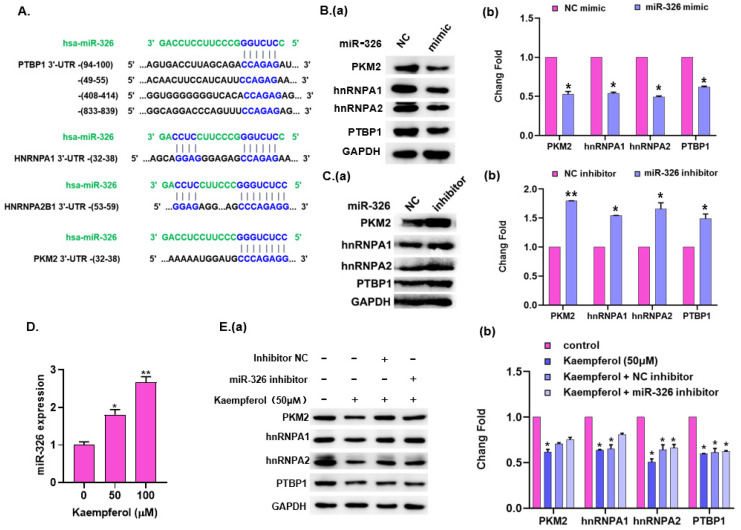
Kaempferol regulates PKM2 expression through miR-326. (**A**) The seed sequence of miR-326 (microRNA-326) matched with the 3′-UTR (untranslated region) of hnRNPA1/A2/PTBP1/PKM2. (**B**) The expression of hnRNPA1/A2/PTBP1 and PKM2 proteins in HCT8-R cells was analyzed, 48 h after miR-326 transfection, by Western blot. (**a**) A representative result from three independent experiments is shown. (**b**) The densitometry analysis of relative protein expression is shown in a histogram. (**C**) Western blot was used 48 h after the transfection of miR-326 inhibitor to analyze the expression of hnRNPA1/A2/PTBP1 and PKM2 in HCT8-R cells. (**a**) A representative result from three independent experiments is shown. (**b**) The densitometry analysis of relative protein expression is shown in a histogram. (**D**) After HCT8-R cells were treated with different concentrations of kaempferol, the expression of miR-326 was analyzed by qPCR. (**E**) HCT8-R cells were treated with kaempferol alone or in combination with miR-326 inhibitor, and the expressions of hnRNPA1/A2/PTBP1 and PKM2 proteins in HCT8-R cells were analyzed by Western blot. (**a**) A representative result from three independent experiments is shown. (**b**) The densitometry analysis of relative protein expression is shown in a histogram. * *p* < 0.05, ** *p* < 0.01.

Considering that miRNA can regulate the expression of PKM2, we then searched in miRNA databases such as Miranda, miRBase and TargetScan; miR-326 attracted our attention because it not only directly targets PKM2, but also indirectly blocks the three heterogeneous nuclear proteins of PTBP1, hnRNPA1 and hnRNPA2. The hypothetical target sequences of miR-326 in these genes’ 3′-UTR sequences are as shown in Figure 5A. To verify this, we next detected the expression of hnRNPA1, hnRNPA2, PTBP1 and PKM2 proteins in miR-326 silenced and miR-326 overexpressed HCT8-R cells. As expected, the overexpression of miR-326 significantly decreased the levels of hnRNPA1, hnRNPA2, PTBP1 and PKM2 proteins (Figure 5B), while down-regulating miR-326 played the opposite role in HCT8-R (Figure 5C). Moreover, we treated HCT8-R cells with different concentrations of kaempferol, and the results show that the level of miR-326 after kaempferol treatment was significantly higher than that of the control group (Figure 5D). It is suggested that kaempferol may inhibit the glycolysis of PKM2 by targeting miR-326, thus increasing the sensitivity of HCT8-R to 5-Fu. To further verify this, we detected the expression of hnRNPA1, hnRNPA2, PTBP1 and PKM2 in HCT8-R cells treated with miR-326 inhibitor and kaempferol alone or in combination (Figure 5E). The results showed that kaempferol alone significantly decreased the levels of endogenous hnRNPA1, hnRNPA2, PTBP1 and PKM2 proteins. Interestingly, kaempferol combined with miR-326 inhibitors significantly reversed that of these four proteins. All these results suggest that kaempferol reversed aerobic glycolysis by directly targeting PKM2 and indirectly blocking PKM mRNA alternative splicing factors, thus increasing the sensitivity of HCT8-R to 5-Fu.

## 3. Discussion

The resistance of tumor cells to chemotherapeutic drugs is one of the main reasons for the failure of tumor chemotherapy. Tumors tend to develop into tumor recurrence and metastasis after drug resistance to chemotherapy, so that 80% to 90% of the deaths of tumor patients are directly or indirectly attributed to drug resistance, which is still one of the biggest challenges in the long-term management of incurable metastatic diseases [17,18]. 5-Fluorouracil, an intravenous synthetic fluorouracil analogue, is currently the most important chemotherapeutic molecule for the treatment of colorectal cancer. By transforming into fluorouracil deoxynucleotides in vivo, it inhibits the activity of thymine synthase, thus interfering with the synthesis of DNA, hindering its repair, inhibiting cancer cell proliferation and starting apoptosis. Although progress has been made in systematic treatment, the 5-year survival rate is still too low [2]. 5-Fu chemotherapy resistance is the main reason for the failure of colon cancer treatment [19,20]. The combination of chemotherapy and chemotherapeutic sensitizers can provide a synergistic therapeutic effect, reduce toxicity and delay the induction of drug resistance. The development of combination therapy constitutes an effective strategy to inhibit cancer cells and prevent drug resistance [6,21,22]. More and more studies have shown that medicine and food homologous plants contain a large number of bioactive substances, such as flavonoids [23], terpenoids [24], polysaccharides [25], polyphenols [26], quinones [27] and so on, which play important roles against tumors and in the reversal of drug resistance. 

Kaempferol (3,4′,5,7 tetrahydroxyflavone) is a yellow tetrahydroxy flavonoid in which four hydroxyl groups are located at positions 3, 5, 7 and 4′. It is mainly derived from the rhizome of Zingiberaceae plant Shannai (*Kaempferol galanga L.*), and it exists widely in many natural products. Its pure products have been extracted from tea, cauliflower, hawthorn, bee pollen, grapefruit and other green plants. In recent years, studies have shown that kaempferol has many nutritional and health effects, such as anti-infection, anti-inflammation, antibacterial, anti-oxidation, prevention and treatment of diabetes and cancer and so on. Kaempferol is considered a potential natural anticancer agent which can fight a variety of cancers in different ways, including breast cancer, liver cancer, gastric cancer and colon cancer [28]. The anti-tumor effects of thirteen phenolic compounds from Tunis papaya Cydonia oblonga Miller have been studied (alone or in combination). The results show that only kaempferol could chemically enhance the sensitivity of 5-Fu-resistant LS174-R cells compared with their parent cells. The phenolic compound combined with 5-Fu has a synergistic inhibitory effect on cell viability and sensitizes tumor cells by affecting the expression of different cytokines [29]. In addition, Li et al. proved that the combination of kaempferol and 5-Fu can inhibit cell viability more effectively than any single drug by inhibiting thymidylate synthase or weakening the activity of p-Akt [30]. In this study, it was found that kaempferol reversed the resistance of colorectal cancer cells to 5-Fu by inhibiting PKM2-mediated glycolysis. Overall, it suggests that kaempferol is a potential chemotherapy agent that can be used alone or in combination with 5-Fu to overcome drug resistance in colon cancer. Importantly, in our present study, we found that administration of kaempferol (100 mg/kg) markedly reduced tumor growth in tumor-bearing nude mice but did not cause any injury to the main organs (heart, liver, spleen, lung and kidney) of the mice (data not shown). However, the clinical trials and possible toxicities of kaempferol in humans have rarely been reported [31]. Resveratrol (RE) is one of the most studied natural phenols. RE has been widely used in clinical research. Although it can provide great health benefits, the adverse reactions of RE to humans have also been reported [32]. It is reported that a 450 mg/d dose of RE is a safe dose for patients weighing 60 kg [33]. However, high doses of RE (2–5g/d) can cause mild diarrhea, nausea and hypersensitivity [32]. We speculate that kaempferol may have the same toxic and side effects as resveratrol; however, further clinical studies are needed.

It is well known that abnormal metabolic changes in cancer cells provide an environment that often promotes tumorigenesis and increases drug resistance [8]. A study has shown that long-term exposure to temozolomide in glioblastoma cells leads to acquired drug resistance, which is related to increased GLUT3, and selective targeting of GLUT3 delays the occurrence of this acquired drug resistance [34]. In addition, the enzymes that directly regulate glycolysis are also related to the promotion of a drug resistance phenotype [8]. Previous studies have pointed out that 3-bromopyruvate, an inhibitor of hexokinase II, can effectively inhibit glycolysis. The inhibition of glycolysis seriously depletes ATP of cancer cells and leads to the rapid dephosphorylation of glycolysis-apoptosis integrator BAD in Ser^112^ and the relocalization of BAX to mitochondria, which can effectively induce apoptosis in multidrug resistant cells [35]. Here, we found that kaempferol could inhibit the expression of PKM2, the key enzyme of glycolysis, and the three splicing factors of the PKM gene, PTBP1, hnRNPA1 and hnRNPA2, thus reversing the resistance of colorectal cancer cells to 5-Fu.

MicroRNA (miRNA) is a kind of non-coding RNA with a short sequence length encoded by endogenous genes. It can regulate gene expression by binding to the 3′-UTR of the target mRNA, thus inhibiting the translation or degradation of mRNA [36]. In recent years, the further study of the mechanism of miRNA has led it to become a new biological target for the prevention and treatment of tumors [37]. Most evidence shows that miRNAs inhibit glycolysis in tumor cells by targeting the alternative splicing of PKM, and miR-374b affects the splicing of PKM1/PKM2 by changing the expression of hnRNPA1, which makes sorafenib-resistant hepatocellular carcinoma cells sensitive to sorafenib therapy [38]. miR339-5p inhibits PKM2-mediated glycolysis by reducing the expression of PTBP1and hnRNPA1 [39]. Most studies have shown that the low expression of miR-326 in most tumors is closely related to poor prognosis, tumorigenesis, metastasis and progression [40]. Our results not only support this view, but also prove for the first time that kaempferol enhanced the expression of miR-326 in colon cancer cells and that miR-326 could inhibit the process of glycolysis by directly targeting PKM2 3′-UTR to inhibit the expression of PKM2 or indirectly block the alternative splicing factors of PKM mRNA, and then reverse the resistance of colorectal cancer cells to 5-Fu. Therefore, this study provides valuable information for the treatment of the 5-Fu resistance of colon cancer with kaempferol.

## 4. Materials and Methods

### 4.1. Reagents and Antibodies

Kaempferol was purchased from Weikeqi (Chengdu, China) and stocked in dimethyl sulfoxide (DMSO) at 200 mM. Fluorouracil (5-Fu, ≥99.0%) was dissolved in double-distilled water (80 mM). In the experiment for kaempferol treatment, the blank control group (0 μM) represented DMSO with a concentration of 0.05%, which was less than 0.1%; its toxicity to cells was thus negligible. DMSO and 5-Fu were purchased from Solarbio (Beijing, China). Antibodies against ABCB1, PKM1, PKM2, hnRNPA1, hnRNPA2 and PTB were purchased from ProteinTech (Wuhan, China). Antibodies for GAPDH, ABCC1 and ABCG2 were obtained from ImmunoWay Biotechnology (Plano, TX, USA).

### 4.2. Cell Culture 

Human colorectal cancer cell line HCT8, 5-Fu-resistant cell line HCT8-R and human normal colorectal mucosal cell FHC were obtained from the Chinese Type Culture Collection (Shanghai, China). The cells were grown in RPMI-1640 medium (GIBCO, New York, NY, USA) supplemented with 10% fetal bovine serum (Biological Industries, Kibbutz Beit Haemek, Israel) at 37 °C in a 5% CO_2_ humidified atmosphere, among which HCT8-R cells were routinely cultured in the medium containing 1 μg/mL 5-Fu. Lipofectamine 2000 (Invitrogen, Carlsbad, CA, USA) was used for transient transfection according to the manufacturer’s protocol. MiRNA mimic and inhibitor were purchased from Genepharma (Shanghai, China).

### 4.3. CCK-8 Assay

The Cell Counting Kit-8 (CCK-8) (Sevenbio, Beijing, China) assay was used to determine cell viability. HCT8 and HCT8-R cells were plated in 96-well plates at a density of 5 × 10^3^/well overnight. Fresh medium containing different concentrations of drugs was added and cultured for the indicated times. Then 10 μL of CCK-8 was added and further incubated at 37 °C for 3 h, and the OD_450_ value was measured using a Bio-Tek MQX200 micro-plate reader (Bio-Tek Instruments Inc., Winooski, VT, USA). The IC_50_ value was calculated with the SPSS software. 

### 4.4. qPCR Assay

Total RNA was extracted using TRIzol Reagent (TAKARA, Kyoto, Japan) based on the manufacturer’s protocol. A total of 1 µg RNA was reverse transcribed to cDNA using the All-in-One First-Strand cDNA Synthesis Kit II for qPCR (with dsDNase) (Sevenbio, Beijing, China). The mRNA expression level was determined using 2× SYBR Green qPCR MasterMix (Sevenbio, Beijing, China). GAPDH was used as the internal control of mRNA expression. The relative expression was quantified according to the 2^-ΔΔCt^ method. The sequences of the primers were as follows: PGP-F: 5′-CCAGAAACAACGCATTGCCA-3′; PGP-R: 5′-GTGCCATGCTCCTTGACTCT-3′; MRP1-F: 5′-ACCCGCTCTGGGACTGG-3′; MRP1-R: 5′-AGCAGACGATCCACAGCAAA-3′; BCRP-F: 5′-CAACTTTCCGGGGGTGAGAA-3′; BCRP-R: 5′-CACTGGTTGGTCGTCAGGAA-3′; PKM2-F: 5′-GCTGCCATCTACCACTTGC-3′; PKM2-R: 5′-CCAGACTTGGTGAGGACGATT-3′; GAPDH-F: 5′-ACCCACTCCTCCACCTTTGA-3′; GAPDH-R: 5′-CTGTTGCTGTAGCCAAATTCGT-3′.

### 4.5. Western Blot Analysis 

After kaempferol treatment, the total protein was prepared from cultured cells and the concentrations were determined using a BCA Protein Assay Reagent kit (Pierce Biotechnology, Rockford, IL, USA). Then equal amounts of protein (50 ng) were separated by 10% SDS polyacrylamide gel electrophoresis (SDS-PAGE) and transferred to polyvinylidene fluoride (PVDF) membranes (Millipore, Boston, MA, USA). After blocking with a rapid blocking buffer (Sevenbio, Beijing, China) for 15 min, the membranes were incubated with the indicated concentration of primary antibodies (PGP 1:1000; MRP1 1:2000; BCRP 1:2000; PKM2 1:1000; hnRNPA1 1:1000; hnRNPA2 1:1000; PTBP1 1:1000; GAPDH 1:2000; Actin 1:2000) overnight at 4 °C, then washed with TBST buffer three times before incubation with the indicated HRP-conjugated secondary antibodies (1:1000) at room temperature for 2 h. Finally, the target proteins were detected using an enhanced chemiluminescence (ECL) substrate reaction kit (Sevenbio, Beijing, China). 

### 4.6. Clonogenic Assay 

HCT8-R cells were seeded into a 6-well plate at a density of 5×10^3^/well and treated with various doses of kaempferol for one week, during which the medium was changed once (contain kaempferol). The colonies were fixed with methanol and stained with 0.1% crystal violet. Then, photos were taken and the number of clones was counted. 

### 4.7. Assessment of Cell Apoptosis

After HCT8-R cells were treated with different doses of kaempferol for 48 h, the apoptosis assay was performed using the Annexin V/PI Apoptosis Detection Kit (BD, USA) according to the instructions. Briefly, the cells were digested with 0.25% trypsin without EDTA and washed with pre-cooled PBS; the cell concentration was adjusted to 10^5^. After the cells were incubated in the dark with 1× binding buffer including 5 μL Annexin V-FITC and 5 μL propidium iodide (PI) for 15 min, the apoptosis was detected using flow cytometry (BD FACSCalibur, New York, NY, USA). 

### 4.8. Glucose Consumption and Lactate Production Analysis

After the cells were treated with kaempferol for 48 h, the contents of glucose and lactate in the culture medium were detected using the Glucose Assay Kit and Lactic Acid Assay Kit (Nanjing Jiancheng, Nanjing, China), respectively. The principle of the glucose test is that glucose in the sample was treated with glucose oxidase to form gluconic acid and hydrogen peroxide, and then hydrogen peroxide coupled 4-aminoantipyrine with phenol under the action of peroxidase to synthesize quinones that could be determined by the spectrophotometer. The contents of glucose in the medium were calculated according to the calculation formula: glucose content (mmol/L) = absorbance value of sample tube (A)/absorbance value of standard tube × standard concentration (5.55 mmol/L).

The principle of lactic acid determination is that acid dehydrogenase (LDH) catalyzes the dehydrogenation of lactic acid to pyruvate, which converts NAD^+^ into NADH. Among them, 5-methylphenazinium methosulfate (PMS) reduced nitrotetrazolium blue chloride (NBT) to purple compounds which could be determined by the spectrophotometer. The contents of lactic acid in the medium were calculated according to the formula: lactate content (mmol/L) = sample tube absorbance value (A)/standard tube absorbance value × standard concentration (3 mmol/L).

### 4.9. Statistical Analysis 

Data from at least three individual experiments are presented as means ± standard deviation (SD). *p* values were calculated with one-way ANOVA and Dunnett’s test, and *p* < 0.05 was considered to be statistically significant.

## 5. Conclusions

We analyzed the drug resistance of 5-Fu-resistant HCT8-R cells and demonstrated that the dietary flavonoid kaempferol enhanced the expression of miR-326 in colon cancer cells; we also showed that miR-326 could inhibit the process of glycolysis by directly targeting PKM2 3′-UTR to inhibit the expression of PKM2 or indirectly block the alternative splicing factors of PKM mRNA, and then reverse the resistance of colorectal cancer cells to 5-Fu.
